# VBM5 vs AIMS65: a comparative evaluation of prognostic scores in upper gastrointestinal bleeding

**DOI:** 10.62838/jccm-2026-0045

**Published:** 2026-10-30

**Authors:** Ilie-Marius Ciorba, Nicoleta Maria Crăciun Ciorba, Paul Grama, Simona Bățagă

**Affiliations:** UMFST G.E. Palade Târgu Mureș; UMFST G.E. Palade Târgu Mureș IOSUD; Gastroenterology Clinic, Mures County Emergency Clinical Hospital

**Keywords:** acute variceal bleeding, cirrhosis, mortality prediction, AIMS65, bootstrap validation

## Abstract

**Background:**

Acute variceal bleeding is a life-threatening complication of cirrhosis in which early mortality risk stratification may support triage, monitoring intensity, and resource allocation. General upper gastrointestinal bleeding scores such as AIMS65 are widely used, but their performance may differ in variceal bleeding, where hepatic dysfunction and organ failure strongly influence outcomes. We aimed to derive and internally validate a parsimonious presentation-based mortality prediction model, VBM-5, and to compare its apparent performance with AIMS65.

**Material and Methods:**

We performed a single-center retrospective cohort study including consecutive adult patients with cirrhosis and endoscopically confirmed acute variceal bleeding admitted between January 2024 and December 2025. The outcome was in-hospital mortality. VBM-5 was derived using logistic regression with five prespecified presentation variables: age, albumin, creatinine, international normalized ratio, and first laboratory hemoglobin. Internal validation used bootstrap optimism correction. Discrimination was assessed using the area under the receiver operating characteristic curve (AUC), and calibration was evaluated using Brier score, calibration-in-the-large, calibration slope, and calibration plots. AIMS65, MELD-Na, Child–Pugh class, and an exploratory VBM-5 bedside points score were evaluated as comparators. Decision-curve analysis was performed as an exploratory assessment of clinical utility.

**Results:**

The cohort included 224 patients, of whom 53 died during hospitalization (23.7%). The continuous VBM-5 model showed higher apparent discrimination than AIMS65 as captured in the available retrospective data (AUC 0.916 vs. 0.829; DeLong p=0.0012). Bootstrap optimism for VBM-5 AUC was 0.014, yielding an optimism-corrected AUC of 0.902; the out-of-bag mean AUC was 0.892. AIMS65 out-of-bag mean AUC was 0.828. The exploratory bedside VBM-5 points score showed a graded increase in observed mortality across score categories. However, comparison with AIMS65 was limited by incomplete standardized availability of systolic blood pressure and mental status, and calibration of mapped comparator scores was cohort-derived.

**Conclusions:**

In this single-center derivation cohort, VBM-5 showed promising optimism-corrected discrimination for in-hospital mortality after acute variceal bleeding. Nevertheless, the positive hemoglobin coefficient, borderline events-per-variable ratio, treatment heterogeneity, and incomplete AIMS65 component ascertainment limit transportability. External multicenter validation, reassessment of coefficient stability, and recalibration are required before clinical implementation.

## Introduction

Acute variceal bleeding is a life-threatening complication of portal hypertension linked to decompensated liver cirrhosis. Early identification of patients at increased risk of death allows the triage of cases (ward versus high dependency versus intensive care), monitoring severity and accurately timed escalation of treatment strategies. AIMS65 (Albumin, INR, Mental status, Systolic blood pressure, age at least 65) is a validated bedside score that predicts in-hospital mortality using five available variables and has been widely adopted in acute upper gastrointestinal bleeding (both variceal and non-variceal) [1, 2, 3, 4, 5].

Prediction tools may be biased to case-mix and setting, and their performance may differ in variceal bleeding, where hepatic failure and multiorgan dysfunction strongly influence outcomes. We therefore compared AIMS65 with a parsimonious cirrhosis-oriented logistic model (Variceal Bleeding Mortality by 5 parameters – VBM-5) based on available laboratory and demographic variables [6,7].

Our objective was the development of a probability model aimed at clinicians for risk stratification of cirrhotic patients with variceal bleeding, along with a pragmatic bed-side score. We compared discrimination of VBM-5 versus AIMS65 for in-hospital mortality and, as a secondary aim, to evaluate internal validity using bootstrap optimism correction and out-of-bag performance estimates [6,8].

## Materials and Methods

We performed a retrospective cohort study of consecutive patients presenting to the Emergency department and admitted with endoscopically confirmed variceal bleeding in the Gastroenterology department of a tertiary referral center for acute gastrointestinal bleeding, between January 1st 2024 and December 31st 2025. Clinical data (medical history, vital signs parameters, hematological markers, liver enzymes, coagulation parameters, renal parameters, albumin), were collected from patient charts and endoscopic findings were collected from the endoscopy logs, checked for correspondence through patient identification markers (chart number, name, age, diagnosis). Laboratory examinations were performed using Sysmex XN-1000 system (Sysmex Europe, Hamburg, Germany) for hematological determination, respectively Cobas Pure e402 system (Roche Diagnostics, Rotkreuz, Switzerland) for the other biochemical markers. Endoscopy was performed by using an Olympus Evis Exera II standard gastroscope (Olympus Corporation, Tokyo, Japan), after getting patient written consent for endoscopy. The report follows STROBE guidance for observational studies and TRIPOD-relevant items for prediction model evaluation [ 9, 10, 11, 12].

### Participants

Our cohort was developed using standardized inclusion and exclusion criteria.

The inclusion criteria consisted of patients of age 18 or over, diagnosed with liver cirrhosis (independent of etiology), with acute upper gastrointestinal bleeding and an endoscopically confirmed diagnosis of variceal bleeding. The exclusion criteria were non-variceal bleeding, incomplete bloodwork, lack of consent for endoscopy, and missing outcome or predictor data (none absent for AIMS65, VBM-5 predictors, or outcome).

The outcome we monitored was in-hospital mortality (death before discharge) derived from the database field Survival.

Baseline characteristics recorded in the construction of the cohort database include demographics, cause and stage of cirrhosis (by standard scores, Child, MELD, MELD-Na where applicable), endoscopic findings, hemoglobin, leucocytes, platelets, TGO, TGP, international normalized ratio (INR), total and direct bilirubin (mg/dL), serum proteins and albumin (g/dL), serum sodium and potassium, creatinine, blood urea, transfusion information and in-Hospital survival. Data was collected for each patient upon admission and after written consent, with attention to bias (sample collection was performed before any volume resuscitation or blood product transfusion was performed).

### Predictors

AIMS65 was analyzed as recorded in the database, with higher scores indicating higher predicted risk. VBM-5 is a five-variable logistic model generating predicted probability of death. In the rationale for this score, we used the outcome ”mortality” and the following predictors (all continuous, objective and available): age (years, scale per 10 years), albumin (in g/dL), creatinine (in mg/dL), International normalized ratio and hemoglobin (in g/dL, first value recorded on presentation, prior to resuscitation) (Table 1).

**Table 1:** VBM-5 selected criteria

**Criteria**	**Reasoning**
Albumin	Measures hepatic reserve, systemic inflammation and nutritional status. Albumin is a crucial severity marker in cirrhosis and appears directly in AIMS65.
International Normalized Ratio (INR)	Represents hepatic failure (coagulation factor production) and correlates directly with advanced portal hypertension. It also provides complementary prognostic information along with albumin.
Creatinine	Reflects renal dysfunction, acute kidney injury — one of the strongest predictors of mortality in decompensated cirrhosis. Adds prognostic power beyond bleeding severity.
Hemoglobin	Stand in for bleeding burden and physiologic compromise at presentation. Provides a practical “bleeding severity” evaluation similar to the Glasgow-Blatchford score.
Age	Shows baseline vulnerability, frailty and potential comorbidity burden. Similar to the “age” domain in Rockall and the general “severity” in AIMS65.

### Rationale

This set gives us direct information on: liver reserve + renal function + bleeding severity + baseline frailty in only five variables. To formulate the score, we used a standard logistic regression: “logit” (p_”death” )=β_0+β_1 (“Age” /10)+β_2 (“Albumin”)+β_3 log^⁡^ (“Creatinine”)+β_4 log^⁡^ (“INR”)+β_5 (“Hemoglobin”)

The developed and internally validated coefficients (on our cohort) were calculated as β0 (Intercept) = −1.0675, β1 (Age/10) = +0.1138, β2 (Albumin g/dL) = −1.500708, β3 (ln Creatinine) = +1.61194, β4 (ln INR) = +3.37686, β5 (Hemoglobin g/dL) = +0.284075 (Table 2). Thus, the linear predictor was computed as: LP = −1.0675 + 0.1138·(Age/10) − 1.500708·Albumin(g/dL) + 1.61194·ln(Creatinine) + 3.37686·ln(INR) + 0.284075·Hemoglobin(g/dL).

**Table 2:** Developed coefficients for VBM-5 calculation

**Predictor**	**β (SE)**	**OR**	**95% CI for OR**	**p-value**
Intercept	−1.0675 (2.2202)	0.344	0.004–26.682	0.630
Age (per 10 years)	0.113 (0.2001)	1.121	0.683–1.497	0.565
Albumin (per 1 g/dL)	−1.4983 (0.4947)	0.224	0.085–0.589	0.002
Creatinine (ln, per e-fold)	1.6121 (0.3750)	5.013	2.404–10.455	0.000
INR (ln, per e-fold)	3.3764 (0.8601)	29.264	5.422–157.935	0.0001
Hemoglobin (per 1 g/dL)	0.2829 (0.1504)	1.327	0.988–1.782	0.0599

Natural-log transformations of INR and creatinine were used because these parameters commonly have nonlinear, “accelerating” risk at higher values. We defined predicted mortality probability: p(death)=1/(1+exp(−LP)).

Furthermore, for bed-side use, we transformed the VBM-5 model from a formula based score to a simple, bedside score with clinically prespecified cutoffs (not data-driven optimization), based on the 5 parameters, with 1 point added for each item set, as follows: Age ≥ 65; albumin < 3.0 g/dL; creatinine ≥ 1.5; INR ≥ 1.5; hemoglobin < 8.0 g/dL (Table 3). Performance of this simplified score is reported separately from the continuous probability model.

**Table 3:** Risk band for VBM-5 cohort

**Predicted risk band**	**n**	**Deaths**	**Observed mortality**	**Mean predicted risk**
<10%	124	4	3.2%	3.8%
10–<20%	24	3	12.5%	16.4%
20–<40%	26	10	38.5%	29.1%
40–<60%	16	9	56.3%	50.1%
≥60%	34	27	79.4%	84.6%

### Statistical analysis

We performed discrimination by ROC AUC for mortality, with death as the positive class. VBM-5 and AIMS65 AUCs were compared using DeLong’s non-parametric test for correlated ROC curves.

Internal validation (VBM-5) was done by bootstrap optimism correction (B=1000). For each bootstrap sample, the VBM-5 logistic regression was refit using the same predictors and transformations while performance was evaluated in the bootstrap sample (apparent) and in the original sample (test). Optimism was estimated as mean (apparent − test). Optimism-corrected AUC and Brier score were computed as apparent minus optimism [6,8,13].

Out-of-bag (OOB) performance: in each bootstrap iteration, AUC was also computed on the OOB observations for both VBM-5 and AIMS65; the OOB mean and 95% percentile interval were reported.

Calibration (VBM-5) was assessed using the Brier score, calibration intercept (CITL) estimated from a model with offset(logit(p)), calibration slope estimated from logistic regression of the observed outcome on logit(p), and a calibration plot combining a LOWESS curve with decile points (Figure 4) [ 6, 7, 14].

**Figure 4.**
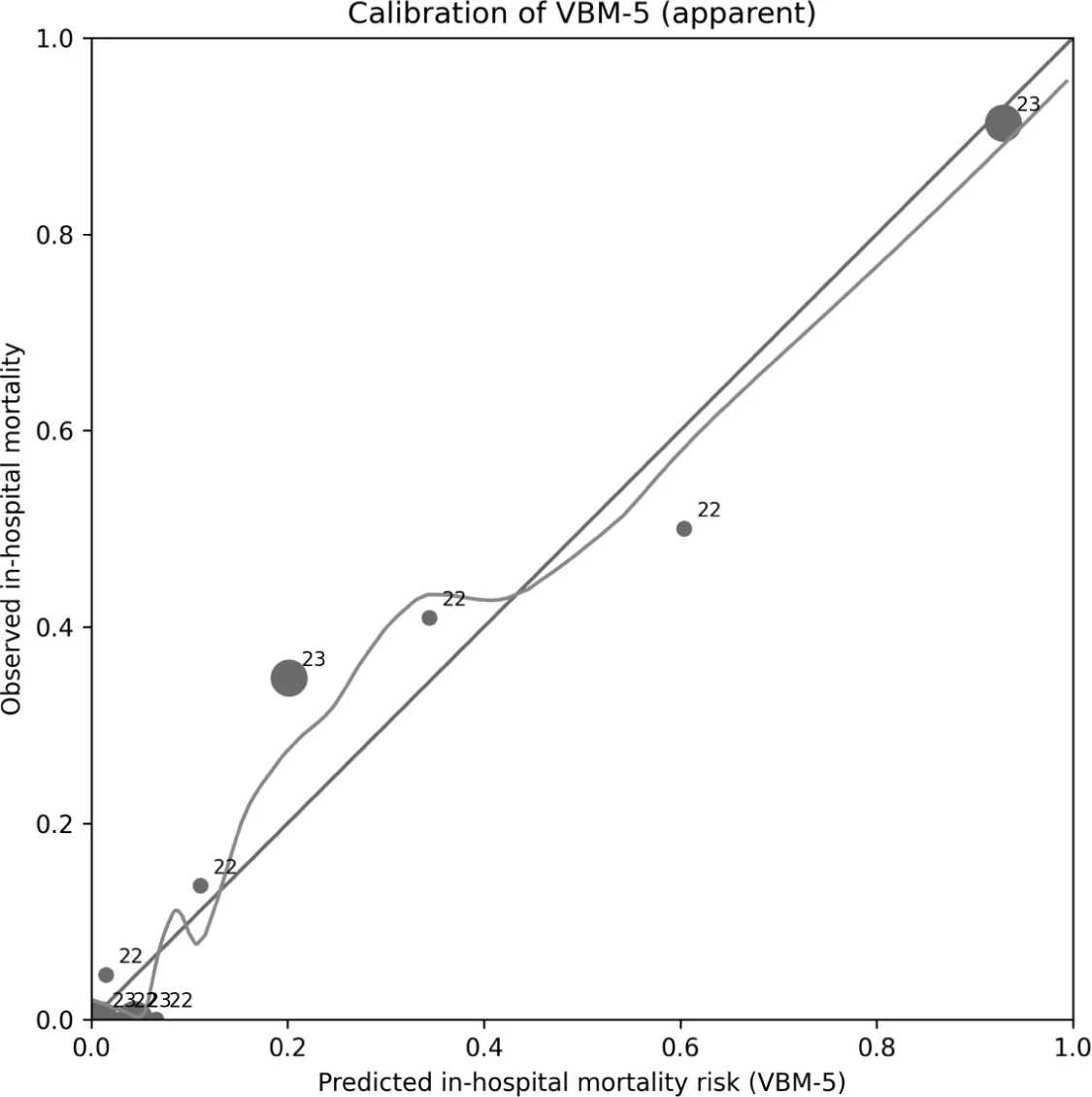
Calibration of VBM-5 for in-hospital mortality (apparent). (n=224; outcome: in-hospital mortality). The diagonal line indicates perfect calibration. The smooth curve is a LOWESS fit of observed mortality on predicted risk. Points represent deciles of predicted risk; each point shows mean predicted probability versus observed mortality within the decile, with point size proportional to decile sample size and labels indicating decile n.

Calibration for AIMS65 and MELD-Na was additionally assessed after logistic mapping with Brier score, calibration-in-the-large and calibration slope computed on the mapped probabilities.

The VBM-5 model code points score (0–5) was evaluated after mapping points to predicted risk through logistic regression (death ~ points) with bootstrap optimism correction (B=2000). Child–Pugh class and MELD-Na score were evaluated as comparator scores using score-to-risk logistic mapping (death /class or score) to obtain predicted probabilities for discrimination and apparent calibration.

Sensitivity analysis (comparator fairness): to reduce the influence of score coarsening, we additionally refit a logistic regression model using the AIMS65 components available in the database (age, albumin, and INR) as continuous predictors (age/10, albumin, ln(INR)); we refer to this as the AIMS-3 model. Systolic blood pressure and altered mental status (the remaining AIMS65 components) were not recorded in a standardized manner in the retrospective database and therefore could not be included. AIMS-3 discrimination was summarized by AUC with a bootstrap percentile interval (B=5000).

Of 834 screened admissions, 610 were excluded for non-variceal bleeding, arriving at a final cohort of 224 patients. There was no missing data for outcome, AIMS65 or VBM-5 predictors, all patients had full workup available, thus complete-case analysis was identical to the full cohort. No patients had to be excluded for lack of data.

Data collection, storage and preliminary analysis of the database was performed using Microsoft Excel. All analyses were performed in R (R Foundation for Statistical Computing, Vienna, Austria, version 4.5.2) using pROC for ROC analysis and DeLong confidence intervals and tests, boot for bootstrap resampling, and ggplot2 for graphical outputs. Discrimination was summarized by the area under the ROC curve (AUC) with 95% confidence intervals (CIs). AUCs were compared using DeLong’s test. Model calibration was summarized by the calibration-in-the-large (intercept) and calibration slope obtained from logistic recalibration of the outcome on the linear predictor and by the Brier score. Calibration plots combined a LOWESS curve with decile points. Internal validation of VBM-5 used bootstrap optimism correction (B=1000), reporting optimism-corrected estimates for AUC, Brier score, calibration intercept, and calibration slope. For AIMS65, out-of-bag (OOB) AUC distributions were obtained analogously from bootstrap resamples [ 6, 7, 8,13, 14, 15].

### Ethics and governance

This study was conducted in accordance with the Declaration of Helsinki and institutional standards for research involving human participants. The protocol was reviewed by the Mureș County Emergency Clinical Hospitals’ Medical Ethics Commission for Clinical studies, approval number Ad 34800 and was granted approval. Because this was a retrospective analysis of routinely collected clinical data with minimal risk to participants, the requirement for informed consent was waived by the reviewing board. All data were de-identified prior to analysis and handled in accordance with applicable data protection policies.\

## Results

### Cohort characteristics

The cohort included 224 patients, with 53 deaths occurring (23.7% mortality). Mean age was 59.7 years (standard deviation 11.4); 174 patients were male (77.7%). There was no missing data in the database so the entire cohort was used for analysis.

**Table 4:** Baseline characteristics of the cohort. (n=224)

**Variable**	**Summary**
Age, mean (SD)	59.7 (11.4)
Male, n (%)	174 (77.7)
Albumin (g/dL), median (IQR)	2.89 (2.50–3.29)
Creatinine, median (IQR)	0.80 (0.65–1.35)
INR, median (IQR)	1.43 (1.23–1.80)
Hemoglobin, median (IQR)	6.75 (6.00–7.90)
AIMS65 score, median (IQR)	2.00 (1.00–3.00)
VBM-5 predicted mortality, median (IQR)	0.08 (0.03–0.34)

### Discrimination and internal validation

Apparent discrimination was higher for VBM-5 (AUC 0.916) than for AIMS65 (AUC 0.829). The AUC difference was 0.087 (DeLong z=3.243, p=0.0012).

In the AIMS-3 sensitivity analysis (refit continuous age, albumin, and ln(INR)), discrimination was intermediate between AIMS65 and VBM-5, with AUC 0.868 (95% bootstrap percentile interval 0.813–0.915). VBM-5 retained higher discrimination than AIMS-3, suggesting that performance differences were not solely due to dichotomization/coarsening of predictors in AIMS65.

Bootstrap optimism for VBM-5 AUC was 0.014, revealing an optimism-corrected AUC of 0.902. OOB mean AUC was 0.892 (95% percentile 0.811–0.954). For AIMS65, OOB mean AUC was 0.828 (95% percentile 0.753–0.897). AIMS65 was not refit within bootstrap resamples (as it is a fixed points score), therefore, these OOB estimates reflect resampling-based precision rather than optimism correction.

Calibration results for AIMS65 and MELD-Na should be interpreted as apparent calibration after logistic mapping fitted in this cohort, rather than transportability to external settings with fixed score-to-risk mappings.

**Table 5:** Performance table. Discrimination and calibration performance. Outcome: in-hospital mortality (n=224).

**Performance metric**	**VBM-5**	**AIMS65**	**Child–Pugh (class)**	**MELD-Na**	**VBM-5 model points**
Sample size, n	224	224	224	224	224
Deaths, n (%)Discrimination	53 (23.7)	53 (23.7)	53 (23.7)	53 (23.7)	53 (23.7)
Apparent AUC (95% CI)	0.916 (0.875–0.957)*	0.829 (0.777–0.881)*	0.805 (0.752–0.855)‡	0.898 (0.851–0.939)‡	0.854 (0.808–0.908)§
Overall accuracy and calibration					
Brier score (95% interval)	0.1067 (0.0811–0.1360)†	0.1383 (0.1129–0.1650)‡	0.1339 (0.1120–0.1585)‡	0.1012 (0.0753–0.1290)‡	0.1273 (0.1012–0.1556)§
Calibration-in-the-large (intercept) (95% interval)	0.007 (−0.484–0.467)†	0.000 (−0.396–0.356)‡	0.000 (−0.390–0.357)‡	0.000 (−0.450–0.394)‡	0.005 (−0.393–0.402)§
Calibration slope (95% interval)	0.899 (0.640–1.155)†	1.000 (0.763–1.336)‡	1.000 (0.699–1.471)‡	1.000 (0.780–1.350)‡	1.001 (0.742–1.312)§

Calibration intercept (CITL) and calibration slope were estimated by logistic recalibration of observed outcome on the model linear predictor (logit of predicted risk). Brier score is the mean squared error of predicted probabilities.

In our cohort we observed a rate of mortality by score: 0: 0/21 (0%), 1: 0/41 (0%), 2: 6/62 (9.7%), 3: 19/56 (33.9%), 4: 19/33 (57.6%), 5: 9/11 (81.8%). Considering that the VBM-5 model outputs a mortality probability, which is useful when translated into risk categories, we defined the following:

**Table 6:** Risk categories and suggested strategies for patient management.

**Risk category**	**Predicted mortality**	**Score**	**Suggested strategy**
Low	<10%	0–1	ward, early endoscopy
Intermediate	10–30%	2	monitored bed / consider ICU
High	30–60%	3–4	ICU-level care / aggressive management
Very high	≥60%	5	

The receiver operating characteristic (ROC) plot (figure 1) compares discrimination for in-hospital mortality between the VBM-5 probability model and the AIMS65 score. Curves closer to the upper-left corner and larger AUC indicate better separation of deaths versus survivors; VBM-5 shows superior discrimination in this cohort (DeLong p=0.0012).

**Figure 1.**
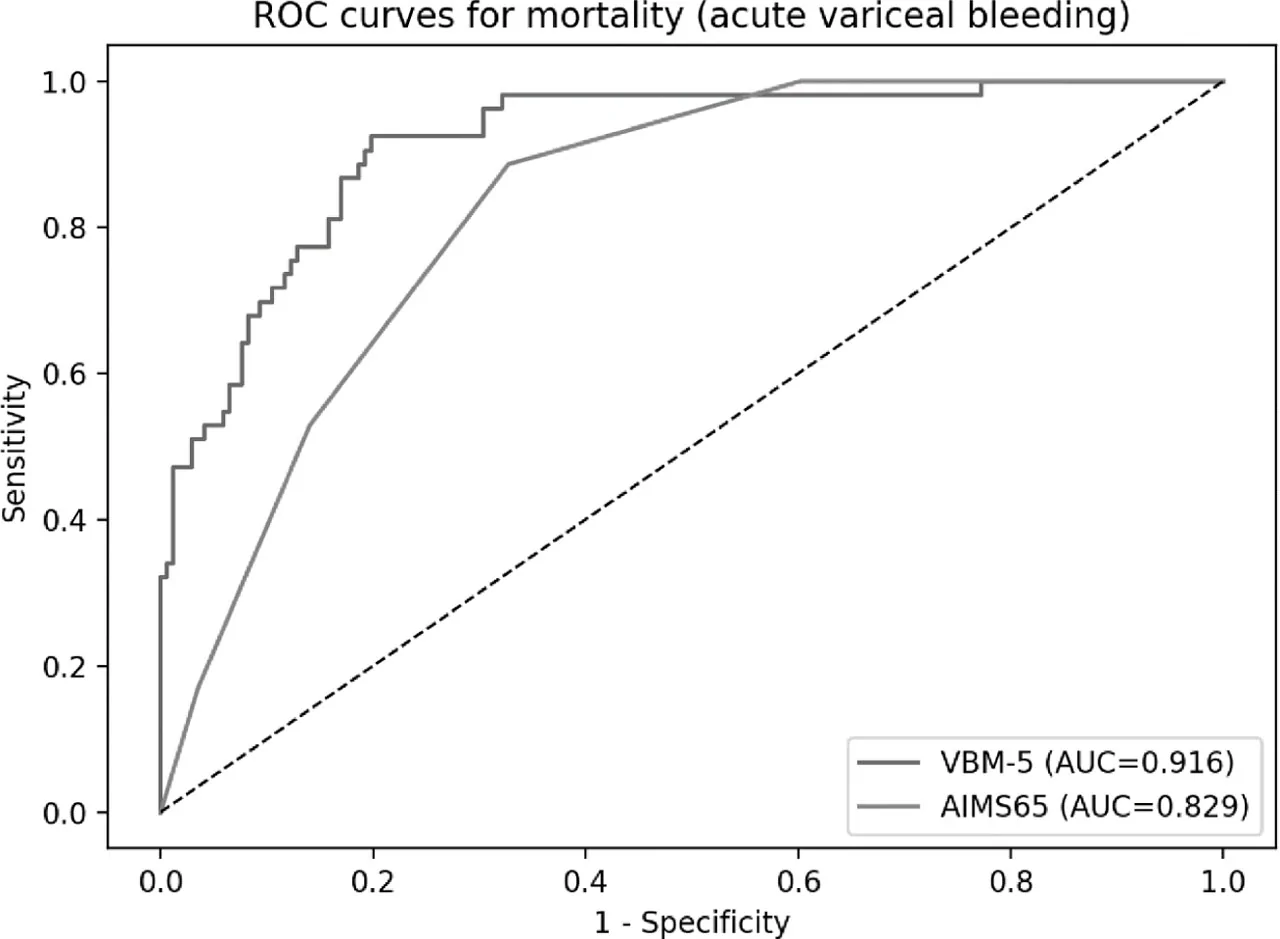
ROC curves for mortality prediction: VBM-5 versus AIMS65. (n=224; outcome: in-hospital mortality)

The bootstrap out-of-bag (OOB) distributions of AUC for VBM-5 and AIMS65 across resamples (B=1000) spread (figure 2) reflects sampling variability, while the relative shift between distributions illustrates the consistent discrimination advantage of VBM-5.

**Figure 2.**
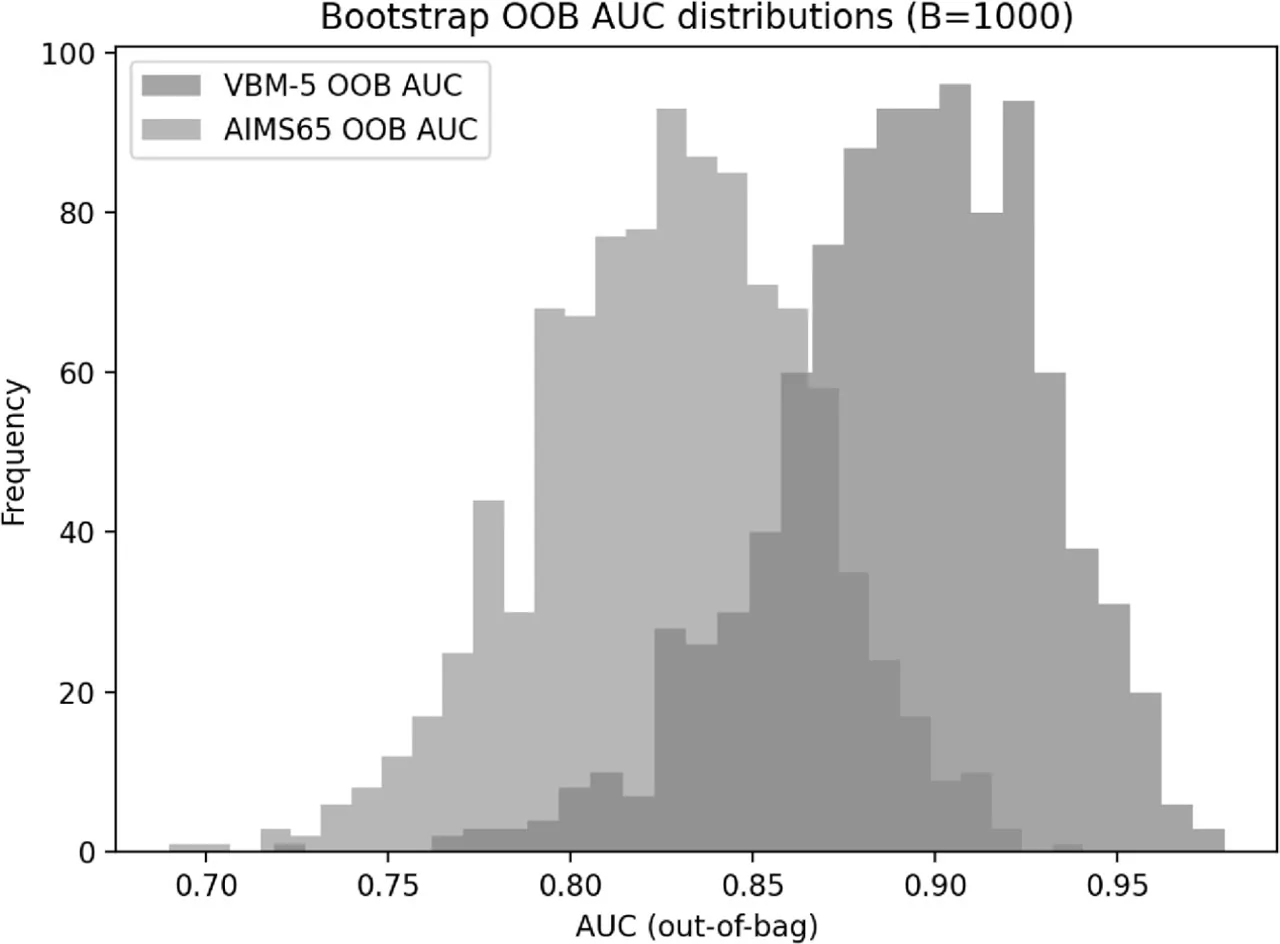
Bootstrap out-of-bag AUC distributions for VBM-5 (B=1000). (n=224; outcome: in-hospital mortality). The dashed line indicates the apparent AUC; the distribution reflects out-of-bag AUC values across bootstrap resamples.

Observed mortality increased with higher AIMS65 scores; the highest proportionate mortality occurred at AIMS65=5, whereas the greatest absolute number of deaths occurred in the AIMS65=3 and AIMS65=4 groups (Figure 3).

**Figure 3.**
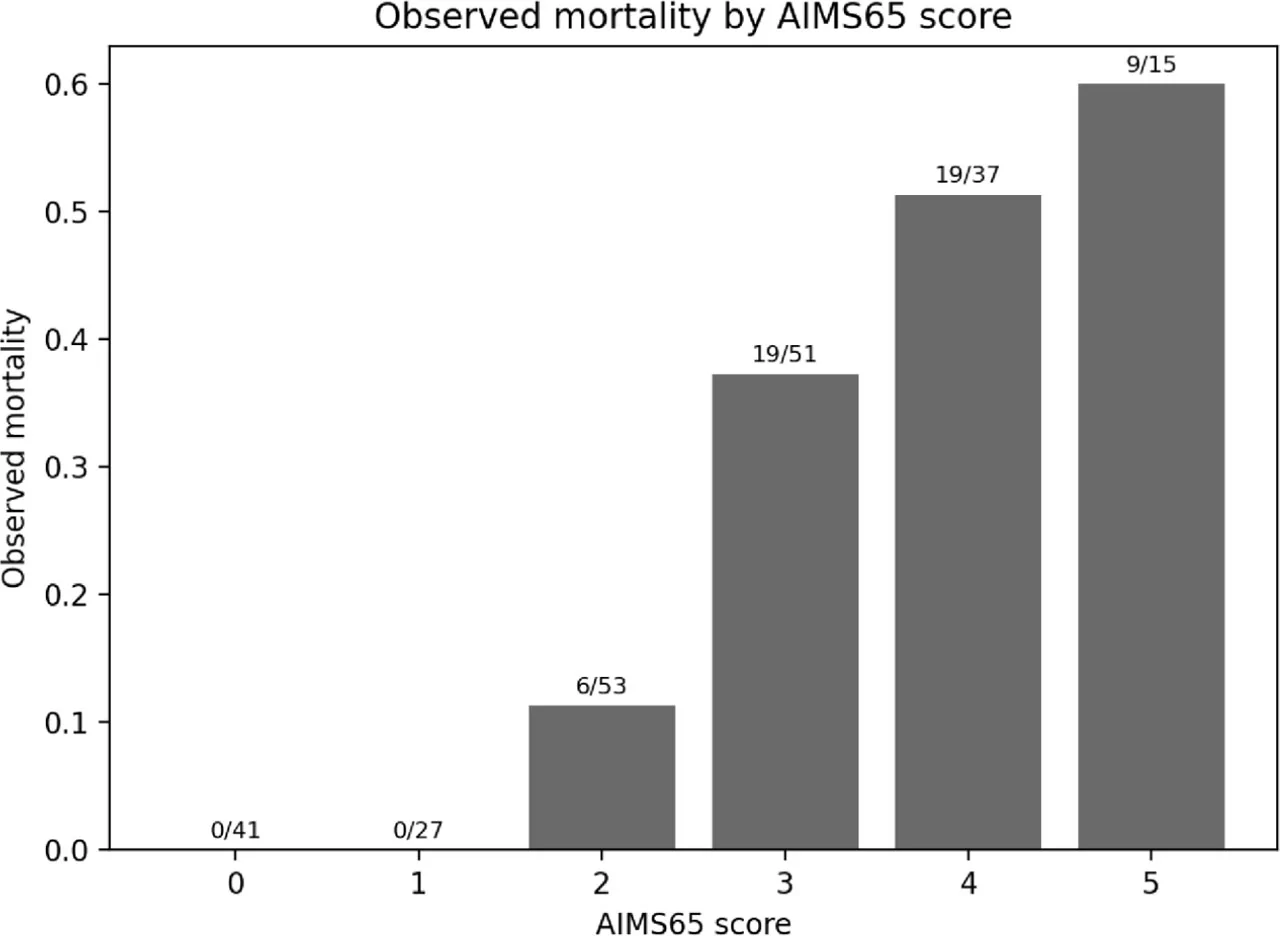
Observed mortality by AIMS65 score (labels show deaths/total). (n=224; outcome: in-hospital mortality).

Predicted risks (figure 4) were computed from the VBM-5 logistic model. Calibration-in-the-large (intercept; ideal 0) and calibration slope (ideal 1) were estimated by logistic recalibration of the observed outcome on the model linear predictor (logit of predicted risk) [6, 7, 14].

Predicted risks (figure 5) were obtained by fitting a logistic model with in-hospital mortality as the outcome and the VBM-5 bedside points score (0–5) as a continuous predictor. Points represent score groups (0–5). Each point shows mean predicted risk versus observed mortality within the group, with point size proportional to group size [6, 7, 14].

**Figure 5.**
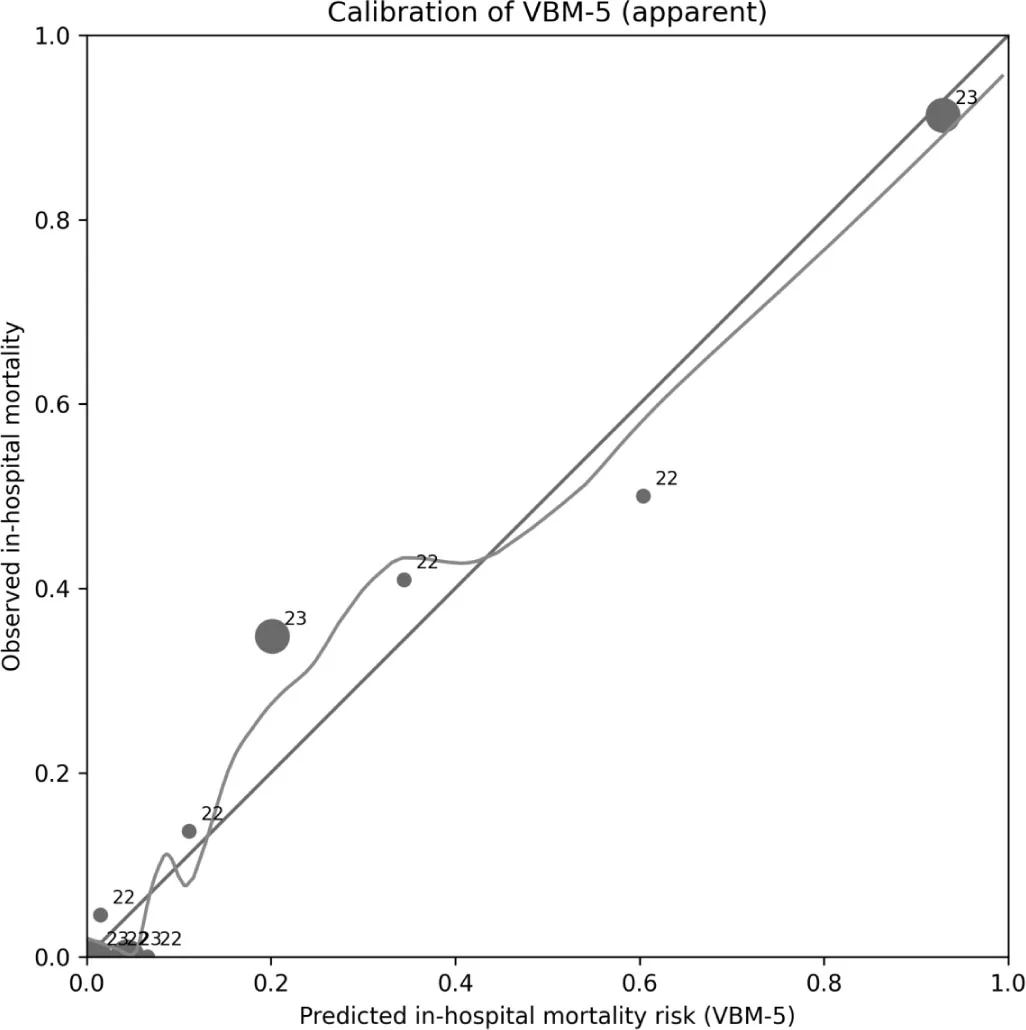
Calibration of the VBM-5 bedside points score after local logistic mapping (apparent). (n=224; outcome: in-hospital mortality). The diagonal line indicates perfect calibration. The smooth curve is a LOWESS fit. Points represent score groups (0–5); each point shows mean predicted risk versus observed mortality within the group, with point size proportional to group size.

**Figure 6.**
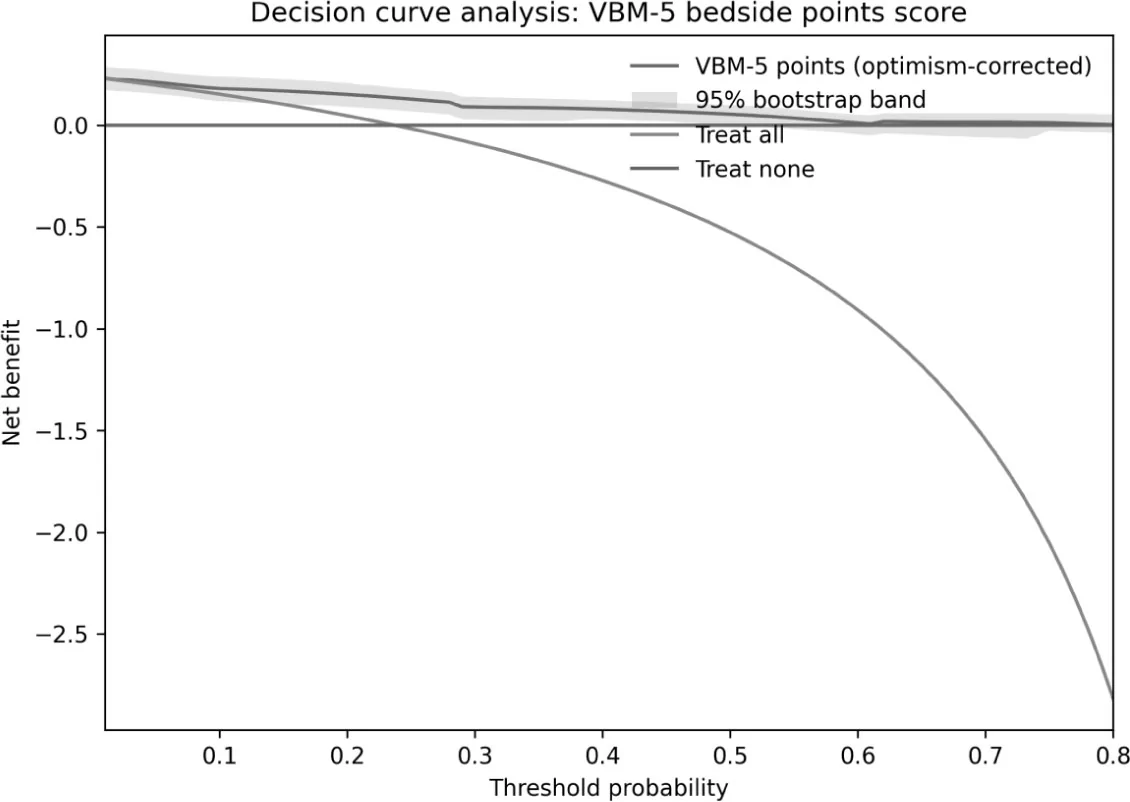
Decision curve analysis of the VBM-5 bedside points score (apparent). (n=224; outcome: in-hospital mortality). Net benefit is shown across threshold probabilities for the points-based model versus treat-all and treat-none strategies.

Net benefit was calculated across threshold probabilities using predicted risks from the points-to-probability logistic mapping. Curves are shown for the optimism-corrected points model, treat-all, and treat-none strategies (figure 6). Shaded bands represent 95% bootstrap percentile intervals of optimism-corrected net benefit (B=500) [14, 16].

Predicted risks were obtained by fitting a logistic model with in-hospital mortality as the outcome and AIMS65 as a continuous predictor (figure 7).

Predicted risks were obtained by fitting a logistic model with in-hospital mortality as the outcome and MELD-Na score as a continuous predictor (figure 8). Points represent deciles of predicted risk (mean predicted probability vs observed mortality), with point size proportional to decile sample size [6, 7, 14].

**Figure 7.**
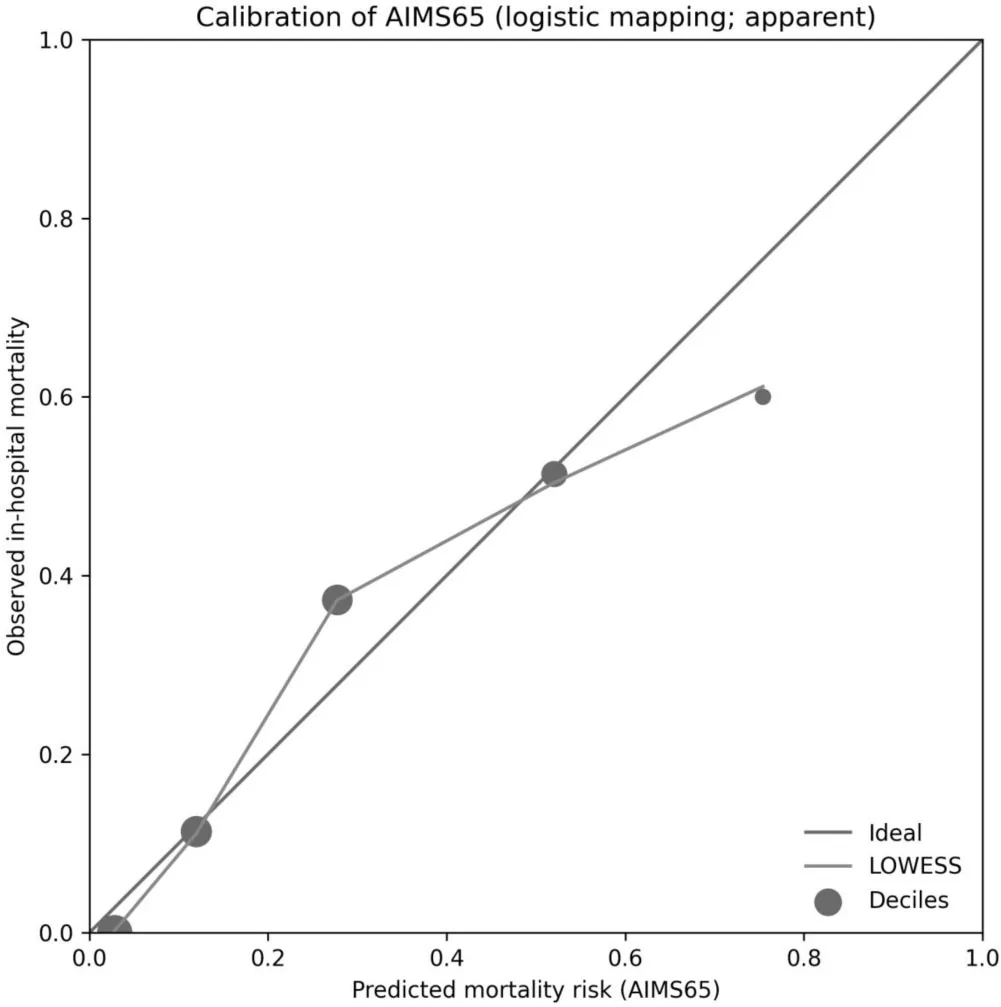
Calibration of AIMS65 for in-hospital mortality after local logistic mapping (apparent). (n=224; outcome: in-hospital mortality). The diagonal line indicates perfect calibration. The smooth curve is a LOWESS fit. Points represent deciles of mapped predicted risk; each point shows mean mapped probability versus observed mortality within the decile, with point size proportional to decile sample size.

**Figure 8.**
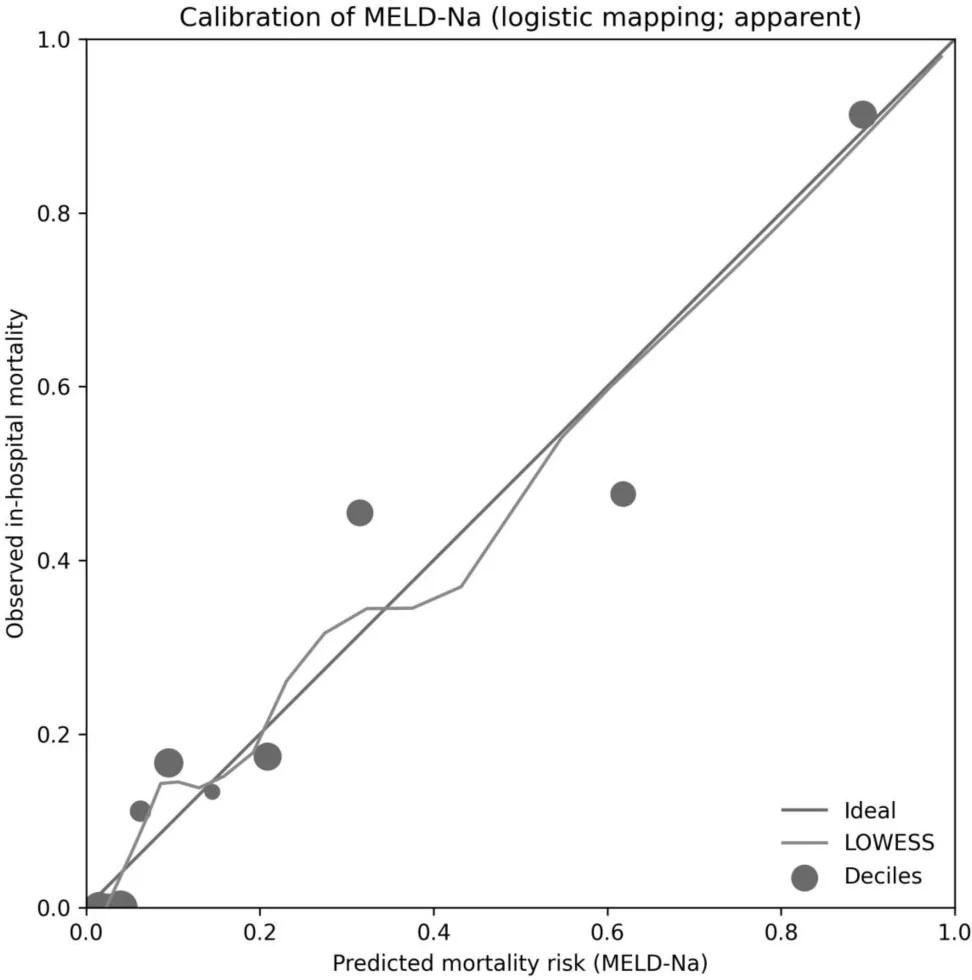
Calibration of MELD-Na score for in-hospital mortality after local logistic mapping (apparent). (n=224; outcome: in-hospital mortality). The diagonal line indicates perfect calibration. The smooth curve is a LOWESS fit. Points represent deciles of mapped predicted risk; each point shows mean mapped probability versus observed mortality within the decile, with point size proportional to decile sample size.

## Discussion

In this cohort composed of acute variceal bleeding cases, the VBM-5 model demonstrated higher discrimination for in-hospital mortality than AIMS65 and remained strongly discriminative after bootstrap optimism correction. These findings support the use of parsimonious cirrhosis-oriented models for risk stratification in variceal hemorrhage when probability outputs are desirable for patient triage and allocation of clinical resources.

Interpretation of the head-to-head comparison with AIMS65 requires caution. While the VBM-5 score is a cohort-fitted continuous probability model, AIMS65 is a fixed bedside points score that intentionally coarsens predictors and includes components such as mental status and systolic blood pressure that may be variably ascertained in retrospective charts. As such, we interpret the observed discrimination difference as performance in this cohort rather than definitive superiority across settings.

Age was prespecified as a predictor based on face validity and prior bleeding scores. In our cohort its conditional effect was modest after adjustment for organ dysfunction markers (albumin, INR, creatinine) and hemoglobin. This illustrates that coefficients reflect conditional associations and may vary with case and population complexity. External validation and potential updating are therefore required.

Although lower hemoglobin was associated with mortality in univariable analysis, hemoglobin showed a positive coefficient in the multivariable model. This reflects the limited ability of a single admission hemoglobin value to quantify the magnitude of recent blood loss and its dependence on timing relative to fluid resuscitation and physiologic equilibration. Hemoglobin is also correlated with markers of hepatic and renal dysfunction and, after adjustment for albumin, INR, and creatinine, the information carried by hemoglobin may capture acute pathology and presentation timing rather than chronic anemia. Importantly, regression coefficients in prognostic models represent conditional associations and should not be interpreted causally. Although hemoglobin was taken from the first in-hospital determination, admission values can still be influenced by timing relative to bleeding start, early fluid administration, and transfusion, which may affect model transportability across systems.

Internal validation indicated moderate optimism in calibration (optimism-corrected slope <1), suggesting that predicted risks may be extreme when applied to new but similar patients or cohorts. As such, the presentation of both apparent and optimism-corrected calibration metrics is crucial and recalibration of the intercept and slope should be considered prior to clinical implementation in other centers or settings with different populations and management protocols.

AIMS65 retained good discrimination in this cohort, consistent with its inclusion of albumin and INR, which reflect hepatic reserve and coagulopathy. Disease-severity comparators (MELD-Na score and Child–Pugh class) provide useful benchmarks but were not designed specifically for hemorrhage episodes. In this context, a bleeding-focused model that produces calibrated probabilities may better support bedside decisions and treatment options.

For clinical use, the VBM-5 model points score showed a clear gradient in observed mortality across score categories and demonstrated net benefit versus treat-all or treat-none strategies across clinically plausible threshold probabilities in decision-curve analysis. These results support further prospective evaluation and need for external validation of both the probability model and the bedside points score, including assessment of recalibration performance. The bedside points score is intended as a rapid triage tool and necessarily sacrifices some discrimination relative to the full probability model (AUC 0.854 vs 0.916 in this cohort). For estimating absolute risk, the VBM-5 probability model should be used; any points-to-risk mapping requires local recalibration.

As the VBM-5 is showing promise as a viable bedside severity score (after external validation and recalibration) in cirrhotic patients with variceal bleeding, with clinical usefulness in the triage, timing and distribution of treatment resources, we are performing further confirmation by prospective evaluation of cases through this score, in parallel with validated, established predictors. The next step for strengthening the practicality of our score is to ask for and encourage external validation by other centers.

This study is retrospective and based on a single-center cohort and therefore may be subjected to selection bias and local treatment-pattern influences (e.g., timing of endoscopy, resuscitation and ICU admission). External validation in independent cohorts is required to establish generalizability and transportability and to quantify the need for recalibration.

The outcome horizon was limited to in-hospital mortality, which is consistent with common bleeding scores but does not capture post-discharge outcomes (such as 30-day mortality or rebleeding). Future studies should, and will, assess longer follow-up horizons and clinically relevant secondary outcomes.

Finally, comparator scores may not have been optimized for this cohort (Child–Pugh class rather than points-based Child–Pugh score), and decision-curve analysis was conducted for the bedside points score. Comparative decision-curve evaluation across all models would require harmonized probability models and prespecified clinical thresholds.

## Conclusions

VBM-5 showed higher discrimination than AIMS65 for in-hospital mortality in variceal bleeding and maintained strong performance after bootstrap internal validation. Calibration findings indicate modest optimism, supporting routine reporting of optimism-corrected calibration and consideration of recalibration prior to use in other centers and different demographic cohorts. The pragmatic VBM-5 bedside points score (based on prespecified cutoffs) demonstrated a clear mortality gradient and favorable net benefit across clinically relevant thresholds, supporting prospective testing and external, multicenter validation before implementation.

## Supplementary Material

Supplementary Material Details
