# Supplementary material for: VBM5 vs AIMS65: a comparative evaluation of prognostic scores in upper gastrointestinal bleeding
Source: J Crit Care Med (Targu Mures). 2026 Oct 30;12(4):686–97. doi: 10.62838/jccm-2026-0045 (PMC13752161; doi:10.62838/jccm-2026-0045)
Supplement: Supplementary file 1 [file jccm-2026-0045-supplementary.pdf]

Supplementary Appendix S1. Hemoglobin directionality in the multivariable VBM-5 mortality model

Objective. To explain the counterintuitive positive coefficient for hemoglobin (Hb) in the multivariable VBM-5 model by (1) contrasting univariable vs multivariable associations, (2) assessing non-linearity using a spline specification and (3) summarizing model performance after removing Hb from the predictor set.

Data and outcome. Analyses use the derivation cohort (n=224) with outcome defined as in-hospital mortality (53 deaths, 23.7%). Predictors are those in the prespecified VBM-5 model: age (per 10 years), albumin (g/dL), ln(creatinine), ln(INR), and hemoglobin (g/dL).

S1.1 Univariable versus multivariable association of hemoglobin

Descriptively, mean admission Hb was lower among non-survivors than survivors (6.53 vs 7.10 g/dL), supporting the expected univariable association of lower Hb with higher mortality.

Table S1.1. Hemoglobin effect estimate in univariable and multivariable logistic regression (outcome: in-hospital mortality).

Model	β (Hb per 1 g/dL)	OR	95% CI	p-value
Univariable (death - Hb)	-0.240	0.79	0.64 to 0.97	0.023
Multivariable VBM-5	0.283	1.33	0.99 to 1.78	0.060

Interpretation. Hb shows a protective univariable association (OR<1), but the direction reverses after adjustment (OR>1). This pattern is consistent with a sign-reversal phenomenon driven by conditioning on strong correlates of global illness severity (albumin, INR, creatinine) and by clinical processes that affect measured Hb (e.g., resuscitation, transfusion timing, hemodilution/hemoconcentration). In this cohort Hb correlated positively with albumin (r=0.32) and negatively with ln(INR) (r=-0.31) and ln(creatinine) (r=-0.23). After regressing Hb on the other VBM-5 predictors, the residual Hb was modestly higher among deaths than survivors (mean residual 0.22 vs -0.07 g/dL), indicating that, conditional on similar hepatic/renal dysfunction markers, relatively higher Hb may proxy unmeasured factors linked to mortality risk.

S1.2 Non-linearity assessment for hemoglobin

We fit an alternative specification replacing linear Hb with a cubic spline basis (B-spline, df=4) while retaining the other VBM-5 predictors unchanged. A likelihood-ratio test comparing spline vs linear Hb showed no evidence of meaningful non-linearity (LR χ^2=2.69 on 3 df; p=0.442). Thus, non-linearity is unlikely to explain the observed coefficient reversal.

S1.3 Model performance after removing hemoglobin

To quantify the incremental value of Hb, we refit the VBM model without Hb (age, albumin, ln(creatinine), ln(INR)). We report apparent discrimination and bootstrap optimism-corrected AUC (300 resamples), along with Brier score. We also tested the nested model comparison using a likelihood-ratio test (1 df).

Table S1.2. Performance of the full VBM-5 model versus a reduced model without hemoglobin.

Model	Predictors	AUC (apparent)	AUC (optimism-corrected)	Brier (apparent)
VBM-5 (full)	age, albumin, lnCr, lnINR, Hb	0.916	0.901	0.097
VBM-4 (reduced)	age, albumin, lnCr, lnINR	0.903	0.894	0.098

The reduction in discrimination after removing Hb was modest (ΔAUC 0.013 apparent; 0.008 optimism-corrected). The likelihood-ratio test comparing full vs reduced models was borderline (LR χ^2=3.63, p=0.057), consistent with limited incremental prognostic information from Hb once albumin, INR, and creatinine are accounted for in this cohort.

Practical implications for interpretation and reporting. Because the adjusted hemoglobin coefficient is sensitive to conditioning on other physiologic and laboratory markers and to the timing of Hb measurement relative to resuscitation and transfusion, hemoglobin should be interpreted as a prognostic marker within the VBM-5 model rather than a causal risk factor. External validation should assess whether the Hb direction and incremental contribution are stable across settings with different transfusion practices and sampling times. If instability is observed, recalibration and/or removal of Hb (VBM-4) could be considered for improved transportability.
